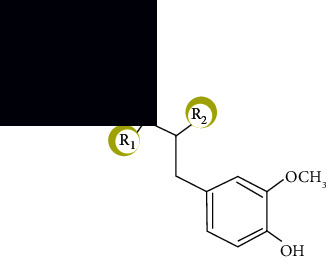# The Role of Bioactive Compounds in Natural Products Extracted from Plants in Cancer Treatment and Their Mechanisms Related to Anticancer Effects

**DOI:** 10.1155/2022/1429869

**Published:** 2022-02-15

**Authors:** Meng Yuan, Guoqing Zhang, Weijun Bai, Xin Han, Chan Li, Siman Bian

**Affiliations:** ^1^Medical Oncology Department of Thoracic Cancer (3), Liaoning Cancer Hospital & Institute (Cancer Hospital of China Medical University), No. 44 Xiaoheyan Road, Dadong District, Shenyang City, 110042 Liaoning Province, China; ^2^Department of Thoracic Surgery, First Affiliated Hospital of Zhengzhou University, Zhengzhou, Henan Province, China 450052

## Abstract

Cancer is one of the greatest causes of death worldwide. With the development of surgery, radiotherapy, and medical agents, the outcomes of cancer patients have greatly improved. However, the underlying mechanisms of cancer are not yet fully understood. Recently, natural products have been proven to be beneficial for various conditions and have played important roles in the development of novel therapies. A substantial amount of evidence indicates that bioactive compounds could improve the outcomes of cancer patients via various pathways, such as endoplasmic reticulum stress, epigenetic modification, and modulation of oxidative stress. Here, we review the current evidence of bioactive compounds in natural products for the treatment of cancer and summarize the underlying mechanisms in this pathological process.

## 1. Introduction

**Figure 1:**
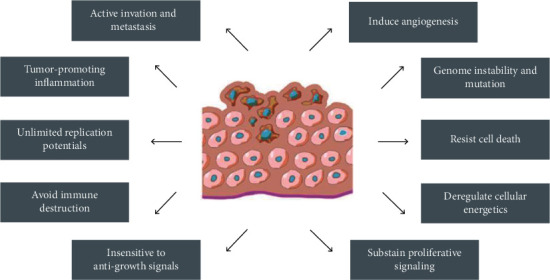
Features of tumors that allow them to grow uncontrollably and metastasize.

Cancer is a pathological condition in which there is unregulated cell growth and cells proliferate in an out of control manner [1]. It continues to be one of the leading killers worldwide. According to the International Agency for Research on Cancer (IARC), a total of 19.3 million new cancer cases and 10.0 million deaths were reported for 2020, and the global cancer burden is expected to be 28.4 million cases in 2040, a 47% rise from 2020. The most commonly diagnosed cancers worldwide are female breast cancer and lung and prostate cancer; the most common causes of cancer deaths were from lung, liver, and stomach cancers [2]. With the increased rates of early detection and treatment, the number of cancer-related deaths has decreased significantly in recent decades. In addition, the current knowledge of molecular and tumor biology has changed the treatment strategies for cancer in the past 15 years. A series of the pathological features of cancer is illustrated in Figure 1. These consist of the biological capabilities of cancer cells to sustain proliferative signaling; insensitivity to antigrowth signals; resistance to cell death; unlimited replication potential, inducing angiogenesis, active invasion, and metastasis; deregulation of cellular energetics; and avoiding immune destruction. Acquisition of the above features depends on the genome instability of neoplastic cells and tumor-associated inflammation, which can provide bioactive molecules to the tumor microenvironment (TME) [3]. The TME is an integrated part of tumors that consists of neoplastic cells such as cancer stem cells, cancer-associated fibroblasts, endothelial cells, pericytes and immune inflammatory cells, the extracellular matrix, cytokines, growth factors, chemokines, and other substances that contribute to the reciprocal heterotypic signaling interactions during the progression of malignancies [4]. These unique characteristics attribute tumor cells possessing the ability of unlimited growth, invasion, and metastasis. Many therapies have been developed that specifically address these properties. However, most current anticancer therapies only provide limited treatment advantages but also result in severe adverse effects [5, 6].

The concept of therapeutic foods has been discussed for many years. A paradigmatic example is that the risks of cancer and cardiovascular disease could be decreased by the Asian and Mediterranean diets [7–9]. A large number of clinical studies have reported that healthy diets rich in fruit and vegetables protect the body against cancer and other diseases. Bioactive compounds are small quantities of food-derived substances that not only provide nutritional value but can also present therapeutic potential. They are found in fruits, vegetables, and whole grains, as well as in many other plants [10]. Bioactive compounds usually contain amino acid residues and can exhibit various physical effects, such as antioxidant, antithrombotic, and antihypertensive activities [11–14]. Many compounds exert more than one of these effects [14–17]. The accumulating evidence indicates that bioactive compounds are gaining credibility for the prevention and treatment of cancer [18, 19]. Recently, the American Institute for Cancer Research (AICR) updated the 26 anticancer foods that are found in the “Global Diet and Cancer Research Report.” We listed these 26 foods and the compounds they contain with respect to their anticancer effects in Table 1, as well as the types of cancer that these foods can fight based on this report. According to the report, strong evidence means there is strong research that shows a causal relationship to cancer, and limited evidence means that the results are generally consistent in their overall conclusions but that the evidence is rarely strong enough to justify recommendations for reducing cancer risk. Moreover, we will discuss the anticancer mechanisms of the compounds in detail in the next section. In summary, dietary habits could prevent the pathological processes of many cancers. In this review, we will first discuss several representative bioactive compounds and then summarize the effects of these compounds on cancer and the mechanisms that are linked with cancer.

## 2. Bioactive Compounds and Their Anticancer Functions

In recent decades, many types of food, including fruits, vegetables, and whole grains, have been determined to have positive effects on human health. Bioactive compounds are the second metabolites of foods and provide health protection in addition to their basic nutritional values [20]. They vary widely in their structures and functions and have the potential to serve as chemotherapeutic and chemopreventive agents in the treatment of cancer [21–24]. In this part, we enumerate the evidence that supports the chemopreventive cancer potentials of these compounds. The bioactive compounds that will be discussed and the food sources of these compounds are presented in Table 2 [25].

### 2.1. Polyphenolic Compounds

Polyphenolic compounds are a diverse group of natural compounds with multiple phenolic functions [26]. These compounds are common in plants and play vital roles in preventing attacks by pathogens and in regulating cell growth. Currently, numerous clinical studies have been carried out to elucidate their positive effects in protecting against cardiovascular diseases, metabolic dysfunction, and aging [27]. With an in-depth knowledge of polyphenols, more research has focused on their anticarcinogenic properties (such as suppressing tumor growth, metastasis, and angiogenesis) [28–30]. Generally, based on the number of phenol rings and molecular structures, the polyphenolic compounds are classified into four subgroups: phenolic acids, stilbenes, lignans, and flavonoids (Table 3).

Food-derived polyphenols usually occur in the form of polymers, esters, and glycosides; when they enter the body, polyphenols are first hydrolyzed by microbes in the intestines and are then absorbed [28]. Subsequently, polyphenols are involved in methylation, sulfation, and glucuronidation [28, 31]. All these forms of polyphenols could be detected in blood but not in natural food. The polyphenol bioactivities depend on various factors, including the rate of absorption, metabolism, and elimination. Additionally, hepatic activity not only can regulate the metabolic activities of polyphenols but can also regulate their delivery to cells and tissues [28]. Dietary polyphenols have been proven to prevent carcinogenesis in diverse biochemical processes [32], such as the inhibition of oxidation, promotion of tumor cell apoptosis, and initiation of immune system function and anti-inflammatory properties [33–35], which will be discussed in detail in the following sections.

#### 2.1.1. Flavonoid Molecules

**Figure 2:**
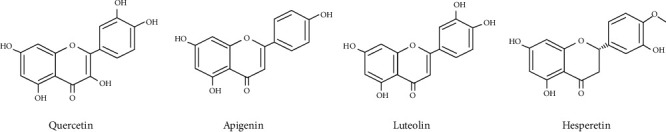
Chemical structure of a few common flavonoids.

Flavonoids are a classic type of polyphenol that are characterized by low molecular weights. Natural flavonoids contain two aromatic rings at both corners and a three-carbon ring at the center, which form oxygenated heterocyclic compounds (Figure 2) [36]. Flavonoids can be synthesized in plants and are not only responsible for the color and aroma of flowers but can also protect plants from various biological and physical attacks by functioning as signal molecules, detoxifying agents, and antimicrobial defensive compounds [25, 37]. Epidemiological, clinical, and animal studies have determined that flavonoids can exhibit strong biological effects against cancer [38–40].

Anthocyanins provide the colors of leaves, flowers, and fruits and protect plants against UV radiation [41]. They can increase the levels of antioxidant, anti-inflammatory, and carcinogen-deactivating enzymes in cells and can inhibit the spread and growth of cancer cells [42]. Flavanols and flavan-3-ols are mainly produced by tea and chocolates. They can influence gene expression and cell signaling and can inhibit the proliferation and metastasis of cancer [43]. For example, EGCG (epigallocatechin gallate), which is mostly obtained from tea, can inhibit proliferation and metastasis, suppress angiogenesis, and induce apoptosis of various types cancer cells [44, 45]. Flavonols are found at high levels in many fruits, vegetables, and grains, among which kaempferol and quercetin are the most representative. Kaempferol inhibits the progression and proliferation of cancer cells and induces apoptosis and DNA damage in cancer cells [46]. It has been explored for possible application in the treatment of breast cancer [47]. The main dietary sources of quercetin are apples, grapes, broccoli, and tea. Quercetin can influence the proliferation of various cancers, such as breast, colon, liver, lung, and gastric cancer [48]. It was recently found that a combination of quercetin and curcumin inhibits Wnt/*β*-catenin signaling and induces cell death [49]. Flavones are mostly found in red plants such as apple, cabbages, carrots, broccoli, and herbs. The most representative compounds are luteolin and apigenin. Apigenin has recently been found to exert anticancer actions by modulating key signaling pathways [50]. Luteolin exerts antioxidative and anti-inflammatory effects and can inhibit the development of cancer cells and induce apoptosis [51, 52]. Fruits and vegetables such as citrus fruits, tomato, and potato are rich in flavanones. They influence gene expression and cell signaling in ways that increase the antioxidant and anti-inflammatory effects. For example, hesperidin inhibits tumor proliferation and induces apoptosis [24]. Recently, naringenin was found to exhibit anticancer effects on breast and skin cancer [53, 54] and the potential ways of enhancing the anticancer effectiveness to achieve the medical application of naringenin have been explored [55]. Derived from soybeans, isoflavones, especially genistein and daidzein, are found to exhibit antioxidant and anti-inflammatory effects and can influence gene expression and inhibit the proliferation and metastasis of cancer cells [56]. A recent study showed that genistein enhances the anticancer effect of cisplatin in cervical cancer cells and can be a potential drug for increasing the activity of chemotherapy [57].

#### 2.1.2. Nonflavonoid Molecules

Resveratrol is the most representative compound of the stilbene family. It is a powerful antioxidant that scavenges free radicals that can damage DNA, and it has been found to inhibit the development and progression of various cancers, such as breast, skin, prostate, colorectal, liver, and lung cancers [58]. Lignans, for example, arctigenin, magnolol, and honokiol, can be mainly found in broccoli, flaxseed, soybeans, and some fruits. They are considered to be important in cancer prevention and management; they can increase the levels of antioxidant, anti-inflammatory, and carcinogen-deactivating enzymes in cell studies; decrease proliferation; and induce the apoptosis of cancer cells [59]. In recent studies, magnolol was found to suppress tumor progression in colorectal cancer [60]. Honokiols can inhibit the invasion, migration, and perineural invasion of pancreatic cancer cells and may be a potential treatment for pancreatic cancer [61]. Phenolic acids can be found in grapes, strawberries, walnuts, green tea, and cereal grains and can be divided into two classes: hydroxybenzoic acid and hydroxycinnamic acid. Phenolic acids exert antiproliferative and proapoptotic effects [62, 63].

### 2.2. Carotenoids

Carotenoids, which are another category of bioactive compounds, are found in various fruits and vegetables [64]. As natural members of the tetraterpene family (e.g., C_40_-based isoprenoid), more than 600 carotenoids contribute to the various visible colors of plants and fruits [65]. These dietary carotenoids are responsible for the yellow, orange, and red colors of fruits, leaves, and flowers [66]. According to the conjugated double bonds, the most common carotenoids in the human body can be divided into *α*-carotene, *β*-carotene, lycopene, lutein, and cryptoxanthin [67–69]. *α*-Carotene and *β*-carotene are usually found in green leafy vegetables and yellow fruits (such as carrots and pumpkins). Tomato is the main source of lycopene. Lutein is also found in green leafy vegetables [70, 71].

The digestion and absorption of carotenoids are a complicated process. First, carotenoids need to be released from foods and form lipid droplets. Due to the hydrocarbon structure, disruption of the droplets or a lack of dietary fat could decrease the bioavailability of carotenoids [72]. In the intestine, bile salts could help decrease their droplet sizes and increase their absorption rates in enterocytes [71]. Some epidemiological studies have indicated that individuals who receive high levels of carotenoids have a lower risk of mortality [73], cancer [74–76], and heart disease [77, 78]. The detailed mechanisms involved in this process include the quenching of free radicals and ROS, inhibiting lipid peroxidation, and regulating cell growth and apoptosis [65]. However, other studies did not observe any effects or even observed harmful effects after the use of carotenoid supplementation [79, 80]. Many factors may account for these findings, such as the baseline characteristics of participants and carotenoid interactions.

Lycopene is a common pigment that is found in red-colored fruits. It has been reported that lycopene is the most effective carotenoid for quenching singlet oxygen [81], and its quenching rate is almost twice as high as that of *β*-carotene. A large amount of evidence indicates that lycopene can provide protection against a broad range of cancers [82, 83].

### 2.3. Sulfur-Containing Compounds

Sulfur-containing compounds contain the chemical element sulfur, which is common in various living organisms. In addition, sulfur is essential for the biosynthesis of various secondary metabolites, which play key roles in the process of protecting against stimuli [84]. Glutathione is the most important sulfur-containing compound in cells. Glutathione is a tripeptide that includes L-cysteine, L-glutamic acid, and glycine [85]. Glutathione is a gamma peptide that links the carboxyl group in glutamate with the *α*-amino group in cysteine. This unusual structure could protect glutathione from being hydrolyzed [86]. In cells, glutathione is present in both reduced and oxidized forms. The ratio of reduced glutathione versus oxidized glutathione will promote the tendency of the reduced form to maintain a reducing intracellular environment and the correct functioning of proteins and enzymes [87]. Numerous studies have reported that glutathione exerts cytoprotective effects in the process of carcinogenesis [88–90].

### 2.4. Terpenoid

As the most abundant secondary metabolites from natural sources, terpenoids are the largest compounds. Derived from mevalonic acid and containing five carbon isoprene units, terpenoids can be found in nearly all living organisms [91]. Many of their metabolites are essential for plant growth and general metabolism [92]. In the past two decades, many studies have focused on their physiological functions. Owing to their diverse biological activities and diverse physical and chemical properties, terpenoids have been employed in anticancer applications since 1990 [93].

### 2.5. Other Compounds with Anticancer Effect

In addition to the bioactive compounds discussed above, other compounds that help to provide anticancer effects should be mentioned briefly. For example, dietary fiber, one of the important nutrients in foods, has many benefits in preventing diseases. Dietary fiber intake can decrease the risk of cancers by diluting carcinogens, disrupting the gut microbiota, and modulating insulin sensitivity and obesity [94, 95]. Vitamin C is a powerful antioxidant. It protects DNA by trapping free radicals and inhibits the formation of carcinogens. It can also regulate the oncogenic cell signaling pathways [96, 97]. Vitamin D modulates the signaling pathways and interrupts the cell cycle by modulating regulators to induce cell cycle arrest and inhibit the progression of cancer cells [96, 98]. Melatonin is mainly secreted by the pineal gland, and it can also be found in foods such as walnuts, cherries, and red wine. Many researchers have found that melatonin exhibits anticancer activity through its antioxidant effects, antiangiogenesis, apoptosis induction, epigenetic alteration induction, and immune function regulation [99, 100].

## 3. Mechanisms in Anticancer Actions of Bioactive Compounds

**Figure 3:**
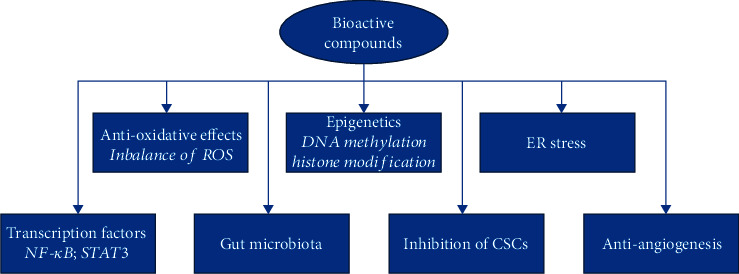
Mechanism of bioactive compounds.

Although the treatment strategies for cancer have developed rapidly in recent decades, the mechanisms of cancer are still not fully understood. Natural bioactive compounds have been proven to be effective in treating cancer. In this section, we will summarize the in vitro and in vivo studies that explore the exact mechanisms of bioactive compounds against different types of cancer (Figure 3).

### 3.1. Antioxidative Effects

**Figure 4:**
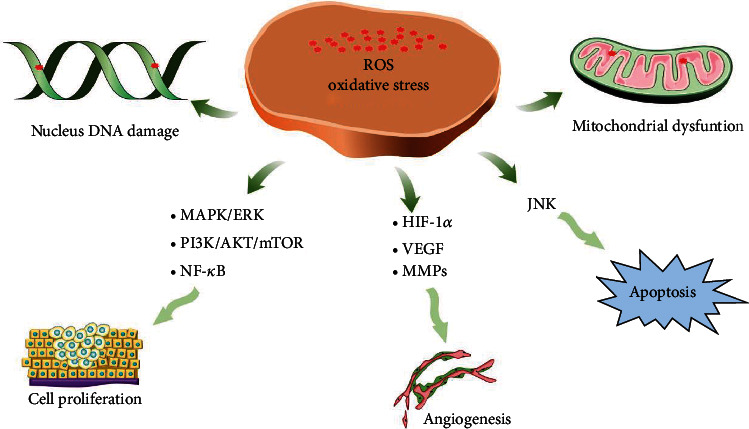
Carcinogenic mechanism of ROS.

Reactive oxygen species (ROS) are a group of reactive chemicals that are derived from oxygen [101], which play vital roles in various physiological and pathological processes, and mainly include hydrogen peroxide (H_2_O_2_), superoxide anion radical (O_2_^·^¯), hypochlorous acid (HOCl), singlet oxygen (^1^O_2_), hydroxyl radical (^·^OH), alkoxyl radical (RO^·^), and the peroxyl radical (ROO^·^) [102]. The main source of ROS is the byproducts that are produced during the mitochondrial electron transport due to aerobic respiration and metal-catalyzed oxidation. An imbalance in ROS levels is identified as oxidative stress, and oxidative stress has a major negative impact on intracellular biomolecules, such as nucleic acids, proteins, and lipids, and results in genomic instability and changes in cell growth (Figure 4) [103].

The intracellular ROS levels are strongly associated with cancer. On the one hand, most cancer cells are characterized by higher ROS levels; in cancerous cell lines, high ROS levels are necessary to maintain rapid proliferation rates due to the Warburg effect. At the initial stage, ROS can cause nuclear and mitochondrial DNA mutations, which induce genomic instability, which are followed by the activation of a series of signaling pathways; this process may promote tumor transformation [104, 105]. In addition, ROS may affect the expression of oncogenes and suppressors [106, 107] via epigenetic modifications [108]. ROS have been reported to be involved in all pathological processes of tumors, and ROS can regulate many proliferation-related genes [109], such as the PI3K/AKT and MAPK/ERK pathways. Tumor-related neovascular formation is another vital step for tumor growth [110]. ROS can promote endothelial cell proliferation and survival [111]. Furthermore, the VEGF pathway can be activated by ROS, which promotes angiogenesis [112]. Regarding metastasis, ROS can activate FAK to enhance tumor cell migration [113], and ROS can activate many proteolytic enzymes, such as MMP and cathepsins, which may facilitate the migration process [114]. On the other hand, the oxidative stress that is due to high ROS levels could also inhibit the cell cycle and eliminate tumor cell growth in a comprehensive model. ROS have been shown to regulate tumor cell death via various pathways [24, 115]. Based on the above evidence, it is now clear that antioxidants can be employed as effective agents for the treatment of cancer by reducing oxidative stress [116, 117].

Many bioactive compounds have been reported to decrease the risk of cancer by participating in the regulation of the redox signaling pathways by modulating the levels of harmful ROS. In an epidemiological study, You and colleagues [118] reported that the organosulfur compounds from vegetables could decrease gastric cancer risk by inducing antioxidant enzymes to increase the cellular antioxidant potential [119]. Sulfur-containing compounds could induce apoptosis of the SH-SY5Y neuroblastoma cell line due to the generation of ROS. The C-Jun N-terminal kinase (JNK) signaling pathway is ROS-dependent [120, 121]. Similar results were also found in human glioblastoma T98G and U87MG cells [122]. Interestingly, the growth inhibition abilities of sulfur-containing compounds are positively related to the number of sulfur atoms [120]. These sulfur-containing compounds can induce G2/M phase cell cycle arrest, which could be abolished by NAC, a type of ROS scavenger, which suggests that cell cycle arrest is ROS-dependent [123, 124]. As free radical scavengers, the polyphenolic compounds were identified to have antioxidant effects decades ago. Their radical-scavenging abilities are also positively related to the number of hydroxyl groups [125]. Some compounds exhibit both antioxidant and prooxidant properties, and the capsaicin-induced apoptosis in cutaneous squamous cell carcinoma cell lines could be diminished in respiration-deficient cells due to ROS generation [126]. Moreover, soy isoflavone generates ROS and induces cell death via its interaction with complex III in the mitochondrial respiratory chain [127].

### 3.2. Epigenetics

Epigenetics consists of a series of heritable changes in gene expression in the absence of changes in the DNA sequences [128]. These modifications include DNA methylation and histone modifications (e.g., acetylation, methylation, and chromatin remodeling). Epigenetics has been reported to be involved in the prevention and treatment of cancer [129]. The current evidence indicates that hypomethylation and hypermethylation within the promoters of tumor suppressor genes are a key feature of cancer, which are present early in the neoplastic process and promote carcinoma expansion [130]. Disruption of histone modifications has been shown to be associated with carcinogenesis by inducing aberrant gene expressions [131]. Chi et al. [132] reported that the disruption of histone methylation caused tumor initiation and progression, while H3L4me3 and H3K27m3 inactivation may exacerbate this process. In addition, the loss of H4K16 acetylation and H4K20 trimethylation is a key marker of cancer [133].

Several bioactive compounds have been reported to possess anticancer potential by influencing epigenetic processes. Folate and vitamin B12 are the main dietary sources of methyl cofactors in the 1-carbon metabolism pathway [134], and choline is an important source of methyl groups for AdoMet synthesis (AdoMet is the key methyl donor in cellular processes). In 1994, Poirier reported [135] that dietary deficiencies of these methyl donors and cofactors may cause the development of hepatocellular carcinoma; in this process, a methyl-deficient diet induced DNA hypomethylation and promoted tumor suppressor gene hypermethylation [136]. The polyphenols could also regulate epigenetic processes to carry out their anticancer functions [137] via promoter methylation and the reactivation of DNA methyltransferase [138]. Histones can regulate chromatin structures and gene expression, and the tails of histones can be modified via acetylation, methylation, phosphorylation, and other modes [139]. Various bioactive compounds can regulate gene expression by altering the posttranslational modifications of histones. Butyrate is one of the first identified anticancer agents to exhibit histone acetylation, and several cell lines have been reported to have inhibitory effects on histone deacetylase (HDAC) activity [140]. In HCT116 human colon cancer cells, butyrate can cause cell growth arrest at the G2 stage and induce apoptosis, which is associated with p21^WAF1^ and Bak overexpression [141]. Lea and colleagues [142] found that in DS19 and K562 leukemic cells, sulfur-containing compounds could inhibit HDAC activity with increased global H3 and H4 acetylation. Dietary polyphenols have also been suggested to be involved with cancer prevention via histone modifications. Genistein could induce p21WAF1 by increasing the acetylation levels of H3 and H4 at the transcription sites in DuPro prostate cancer cells [143]. As for carotenoids, a number of studies have confirmed its regulation for epigenetic processes. Moody et al. had thoroughly described the relationship between lycopene and DNA methylation [144], in which they also indicated that lycopene may exert and alter DNA methylation through systemic antioxidant effect because study has shown that lycopene levels are negatively correlated with oxidative stress [145]. Furthermore, an inverse correlation between lycopene intake and methylation of a CpG in the paraoxonase1 (PON1) promoter had been demonstrated [146]. An in vitro study observed glutathione S-transferase pi 1 (GSTP1) promoter demethylated in lycopene-treated MDA-MB-468 breast cancer cells [4]. The same study reported demethylation of retinoic acid receptor *β* (RARB2) and secretoglobin family 3A member 1 (SCGB3A1) following lycopene treatment. However, Liu and Erdman demonstrated that lycopene and apo-10′-lycopenal are not effective demethylating agents of GSTP1 in the human LNCaP cell line [147]. A similar study also shows that lycopene treatment did not demethylate the GSTP1 promoter or increase GSTP1 expression in such cell line [148]. These results suggest that the effect of carotenoids on epigenetic processes is tumor-specific. Sulfur-containing compounds are essential for the inhibition of cancer via epigenetic changes. Chhabria et al. [149] have shown that allicin can suppress pancreatic cancer cell (MIA PaCa-2) growth via affecting the level of acetylation of H3 lysine 14 (H3 K14), phosphorylation of H3 Ser 10 (H3 S10), and monomethylation of histone H3 lysine 9 (H3 K9). Diallyl disulfide and sulforaphane have also been identified with histone acetylation; several cell lines have reported its inhibition effects on histone deacetylase (HDAC) activity [140]. Lea and colleagues [142] found that in DS19, K562 leukemic cells, and human breast cancer cells, diallyl disulfide and structurally related molecules could inhibit HDAC activity with increased global H3 and H4 acetylation. Terpenoid is another kind of bioactive compounds that may have influences on epigenetic processes. CYP1A1 is important in the bioactivation of procarcinogens. Studies have shown that d-limonene can inhibit, even slightly, the 7-ethoxyresorufin O-deethylase activity of CYP1A1, which may explain its antitumor mechanism [150]. Interestingly, epigenetic processes can also influence the production of active substances in microorganism. H3K4 trimethylation has demonstrated that terpenoid was greatly enriched compared to that of the wild type in Colletotrichum higginsianum [151]. However, similar results have not been confirmed in animal studies. Ozden et al. have assessed the gene-specific DNA methylation in response to d-limonene exposure in rat liver and kidney, and no change of the methylation status of various genes such as c-myc, Cx32, Igfbp2, e-cadherin, VHL, and p15 was observed [152]. Furthermore, a study revealed that the natural mixture of bioactive compounds rather than limonene alone demonstrated anticancer effects in breast cancer through in vivo and in vitro analyses [153]. Many studies have found that bioactive compounds modify the epigenetics in a dose-dependent manner. Therefore, in the future, it will be necessary to determine the effective doses and concentrations of these compounds in relation to cancer prevention or treatment.

### 3.3. ER Stress

The endoplasmic reticulum (ER) is vital for protein synthesis, cholesterol biosynthesis, and calcium signaling [154]. ER environmental homeostasis is crucial for maintaining intracellular physiological functions. Extracellular stresses, such as hypoxia, ROS, and nutrient deprivation, could induce disturbances in ER homeostasis and lead to impaired ER protein-folding environments, which would result in the accumulation of unfolded proteins in the ER, which is called ER stress (ERS) [155]. Various studies have indicated that ERS is associated with cancer, and many ERS-related proteins, such as glucose-regulated protein 78 (GRP78)/binding protein (BiP), activating transcription factor 6 (ATF6), and inositol-requiring protein 1 (IRE1), are involved in many types of cancer [156, 157]. When ER stress occurs, cells initiate the unfolded protein response (UPR), which is a conserved signaling pathway, to maintain ER hemostasis by degrading unfolded proteins for survival. The UPR signaling pathway consists of three typical branches, which are mediated by PERK (protein kinase RNA-like ER kinase), IRE1, and ATF6. When experiencing ER stress, GRP78 dissociates from ATF6. ATF6 translocates to the Golgi apparatus, where it is cleaved by proteases to form ATF6f (the cytosolic ATF6 fragment), which translocates to the cell nucleus and activates the transcription of ERSE (ER stress element), including ER chaperones such as Bip, GRP 94, and CHOP (C/EBP-homologous protein). In addition, it can also activate the transcription of XBP1 (X box-binding protein 1) and ERAD (ER-associated protein degradation) genes [158, 159]. The IRE1 signaling pathway is the second modulator of ER stress signaling. The luminal domain of IRE1 dissociates from GRP78 under ER stress conditions, which causes the phosphorylation and dimerization of IRE1, which activates endoribonuclease and forms mature XBP1. Mature XBP1 regulates ERAD, ER chaperones, and the protein transport and lipid synthesis processes to maintain homeostasis in the ER environment [160, 161]. PERK is the main protein that is responsible for the attenuation of mRNA translation under ER stress conditions and prevents newly synthesized proteins from entering the ER lumen. During ER stress, PERK dissociates from GRP78, which initiates autophosphorylation and specifically phosphorylates eIF2*α* (eukaryotic initiation factor 2). The phosphorylation of eIF2*α* causes it to lose the ability to initiate protein translation and downregulate the level of protein synthesis. In addition, eIF2*α* can promote the selective translation of mRNA encoding ATF4 (activating transcription factor 4). ATF4 upregulates CHOP and induces the expression of various genes, such as those for apoptosis and autophagy [162, 163].

However, under persistent ER stress, the UPR switches to autophagy or to the cell death pathway. CHOP, also known as GADD153 (growth arrest and DNA damage-inducible protein), is encoded by the DDIT3 (DNA damage inducible transcript 3) gene and is one of the most important regulatory genes among the ER stress-induced apoptotic genes and can induce the expression of transcriptional targets such as GADD34, DR5 (death receptor-5), and ERO1*α* (endoplasmic reticulum oxidase 1*α*). ERO1*α* increases ROS production and the outflow of Ca^2+^ from the ER mediated by IP3R (inositol-1,4,5-triphosphate receptor) and disrupts mitochondrial function, which leads to CHOP-dependent cell apoptosis. The activation of GADD34 promotes the dephosphorylation of eIF2*α*, which results in regaining protein synthesis, which thus initiates apoptotic pathways [164–166]. Another possible mechanism by which CHOP induces apoptosis is a decrease in antiapoptotic Bcl-2 expression and increase in BIM (Bcl2-interacting mediator of cell death), which leads to BAX- (Bcl-2 associated X protein-) and BAK- (Bcl-2 antagonist/killer 1-) dependent cell apoptosis. Caspase-12 is located on the endoplasmic reticulum and binds with TRAF2 (TNF receptor-associated factor 2). When ER stress persists or is aggravated, caspase-12 dissociates from TRAF2, is activated, and then activates apoptotic effector enzymes such as caspase-9 and caspase-3, which leads to cell apoptosis. IRE1*α* stimulates the activation of ASK1 and induces the activation of JNK (c-Jun-N-terminal kinase) and p38. Bcl-2 and BIM can be inhibited or activated, respectively, by JNK phosphorylation. p38 can be phosphorylated and activate CHOP [167–169]. Based on this, one therapeutic rationale for cancer is to focus on reducing the accumulation of unfolded proteins [170]. A polyphenolic compound treatment of NCI-H1460 cells could increase the expression of CHOP and GRP78 due to the release of intracellular calcium [171]. Similar results were found for NCI-H1460 cells after terpenoid treatment [172]. Additionally, A549 and 95-D cells treated with terpenoids could induce an increase in CHOP [173]. Saxifragifolin D is a terpenoid, and in MCF-7 and MDA-MB-231 cells, a treatment with saxifragifolin D may induce ERS by disturbing the cytoplasmic calcium and ROS levels [174]. As a kind of polyphenolic compound, curcumin could induce ERS-mediated apoptosis not only in colorectal cancer but also in gastric cancer [174]. The accumulated evidence indicates that natural bioactive compounds exert anticancer effects via ERS; however, most studies have only focused on cancer-related cell lines. In the future, more in vivo studies may be essential to verify the potential effects of these compounds.

### 3.4. Transcription Factors

Chronic inflammation plays a vital role in both the initiation and development of carcinogenesis [175, 176]. Many steps of tumors, such as cellular transformation, proliferation, and metastasis, are related to chronic inflammation. The targets of inflammation provide potential therapeutic pathways for tumor treatments. Among the various molecular targets, transcription factors have gained increasing attention [177]. Currently, a majority of bioactive compounds have been identified to modulate transcription factors in the treatment of cancer [5].

NF-*κ*B is a proinflammatory transcription factor that regulates the expression of various genes that are involved in tumor cell proliferation [178, 179]. Curcumin is reported to inhibit melanoma cell proliferation via NF-*κ*B inhibition [180, 181]. In addition, by blocking NF-*κ*B translocation and binding with DNA, curcumin inhibited the growth of human papilloma virus-induced cervical cancer [182, 183]. Celastrol could inhibit the proliferation of hematopoietic cancer cells via deactivation of the NF-*κ*B/Notch 1 pathway [184]. STAT3 is a transcription factor that is involved in tumor cell proliferation and metastasis. After phosphorylation, STAT3 can translocate to the nucleus and initiate the inflammatory process [185–187]. Curcumin could decrease STAT3 activation and the downstream transcription process [23], which could inhibit pancreatic cancer cell proliferation. In addition, curcumin could also inhibit cyclin D1 via STAT3 Y705 in colorectal carcinoma [188–190]. There are many other transcription factors, such as HIF-1*α*, PPAR*γ*, Nrf2, and FoXM1, that have been reported to be regulated by bioactive compounds in cancer cells [191–194].

### 3.5. Modulation on Gut Microbiota

There are more than 100 trillion microbes from nearly 1000 species in the human GI tract, among which the beneficial gut microbiota helps the human body to maintain the integrity of the intestinal mucosal barrier and to digest fibers and bioactive compounds [195]. The gut microbiota is also important to the homeostasis of the human immune system. Many factors can influence the composition and abundance of the gut microbiota, such as aging, dietary components, and antibiotics, among which diet is the most influential and modifiable. A healthy diet with a variety of foods that are rich in dietary fiber and bioactive compounds can cause the gut microbiota to be more balanced [196]. Diets rich in fruits, vegetables, and whole grains are negatively associated with the risk of cancer. Bioactive compounds, such as polyphenols, have the ability to regulate the balance and composition of the gut microbiota and make an important contribution to protecting the human body [197–199]. Oral probiotics observably decrease the size of liver tumors in C57BC/6N mice by 40% [200]. Moreover, the actions of polyphenols on cancer inhibition are often relevant to the gut microbiota [201].

The gut microbiota-mediated metabolisms of bioactive compounds can also benefit the human body and help to prevent cancer. Bioactive compounds are transformed to metabolites by gut microbiota in the intestine and inhibit angiogenesis and inflammatory factors such as tumor necrosis factor-a (TNFa) and IL-6, which are related to cancer prevention [202]. Many metabolites with greater pharmacological activity may then undergo enzymatic cleavage by methylation, glucuronidation, glycination, or sulfation in liver cells, which are transformed into tissues and excreted into the intestinal tract. Glucuronides are converted to aglycones by gut microbiota and are reabsorbed in the colon and therefore prolong the retention times of bioactive compounds in the body. These compounds can regulate cell division and apoptosis by clearing pathogenic gut microbes and reducing oxidative DNA damage and proinflammatory mediators [203]. It has been indicated that the gut microbiota plays an important role in gastric cancer [204, 205]. Moreover, bioactive compounds could prevent and treat some kinds of cancers by regulating the gut microbiota, such as colorectal cancer, liver cancer, and breast cancer [45].

The gut microbiota is also associated with chemotherapy and immunotherapy for cancer. The reciprocity between the gut microbiota and human body may affect drug pharmacokinetics [206]. Immune checkpoint inhibitors (ICIs) have provided breakthroughs in cancer patients and have been widely used in cancer therapy in the past decade, including the programmed death 1 receptor (PD-1), programmed cell death receptor ligand 1 (PD-L1), and cytotoxic T lymphocyte-associated antigen-4 (CTLA-4) blockade. It has been indicated that the gut microbiota is closely associated with immunotherapy [202]. A metagenomic analysis of fecal samples from patients receiving PD-1 blockade treatment for non-small-cell lung cancer (NSCLC) and renal cell carcinoma (RCC) showed that those patients who failed to respond to PD-1 blockade had lower levels of Akkermansia muciniphila, which had been confirmed to facilitate the recruitment of CCR9+, CXCR3+, and CD4+ T lymphocytes via the IL-12 pathway [207]. The gut microbiota can also influence the efficacy of CTLA-4 blockade. The effects of CTLA-4 blockade are related to Bacteroides species, and tumors in germ-free or antibiotic-treated mice show no response to CTLA-4 blockade [208, 209].

### 3.6. Inhibition of Cancer Stem Cells

Cancer stem cells (CSCs) have been confirmed to have an important role in the development of cancer. Researchers have isolated CSCs from nearly all solid tumor cell populations. CSCs have the biological characteristics of self-renewal, multidirectional differentiation, and infinite proliferation and can also exhibit resistance to chemotherapy drugs, tumorigenicity, and strong invasive and metastatic abilities at the same time [45]. The irregular activation of signaling pathways such as Wnt/*β*-catenin, Notch, and Hh of cancer stem cells may influence the capacity for self-renewal and promote tumor initiation, invasion, and metastasis. Many studies have suggested that bioactive compounds such as polyphenols and flavonoids can inhibit the propagation of cancer and are promising drugs that target CSCs [203]. Sulforaphane can inhibit Wnt signaling and downregulate epithelial-mesenchymal transitions [210]. EGCG was effective in inhibiting self-renewal and the expression of pluripotency-maintaining transcription factors in human CSCs [44, 211]. Cinnamic acid can reduce the levels of CSC markers in HT-29 colon cancer cells [212]. Retinoic acid derived from vitamin A can downregulate Notch signaling and cause CSCs to differentiate or decelerate their processes [213]. Matcha can convert cancer cells to a resting state and inhibit the proliferation of breast CSCs by downregulating mitochondrial proteins and glycolytic enzymes and inhibiting mTor signaling [214].

### 3.7. Antiangiogenesis

Angiogenesis is crucial for the progression and prognosis of all types of cancer. Vascular endothelial growth factor (VEGF), which is a cytokine produced by tumor cells, plays a key role in angiogenesis. Therefore, an anticancer therapy was developed by inhibiting VEGF or other related signaling pathways to block the supply of energy. Bevacizumab, a humanized anti-VEGF monoclonal antibody, is now widely used to treat many kinds of cancer. However, more effective drugs should be found to achieve more accurate therapies and address drug resistance [215].

Interestingly, diet-derived phytochemicals have been reported to suppress angiogenesis and have recently been expected to represent promising anticancer therapies [21, 22, 216]. The prodrug of EGCG could inhibit phosphoinositide 3-kinase (PI3K)/Akt/mammalian target of rapamycin (mTOR)/hypoxia inducible factor-1 *α* (HIF-1*α*) pathway by decreasing the secretion of vascular endothelial growth factor A (VEGFA) in endometrial cancer cells in xenograft models and thus suppress cancer angiogenesis [217], and it is also reported to downregulate the chemokine (C-X-C motif) ligand 12 (CXCL12) in stromal cells by pro-EGCG treatment and to restrict the migration and differentiation of macrophages and thereby inhibit the infiltration of VEGFA-expressing tumor-associated macrophages (TAMs), which thereby inhibits TAM-secreted VEGFA in endometrial cancer [218]. Polysaccharides from Korean citrus hallabong peels inhibited angiogenesis by decreasing the tube formation of human umbilical vein vascular endothelial cells and suppressed the cell migration of MDA-MB-231 cells via downregulation of MMP- (metalloproteinase-) 9 [219]. Moreover, nobiletin from citrus fruit peel is also suggested to suppress angiogenesis and metastasis in CRC via the inhibition of MMP and VEGF [220]. Delphinidin from Punica granatum is an anthocyanidin that exhibits antitumor activities. Delphinidin shows antiangiogenic activity by suppressing EGF-induced VEGF protein production and HIF-1*α* expression [221]. Luteolin, a bioactive flavone, exhibits anticancer activity by inhibiting VEGF production and by blocking VEGF binding with the receptor in breast cancer [222]. Moreover, it is also reported to suppress vasculogenic mimicry formation and inhibit VEGF [223]. Curcumin has been considered to suppress gastric carcinoma proliferation by inducing apoptosis in tumor cells and by inhibiting the STAT3, VEGF, and HIF1*α* signaling transduction pathways [224]. Oxyresveratrol inhibits VEGFR-3, VEGFC, and CD31, which provides protection against cancer growth [225]. 3-O-Acetyloleanolic acid from Vigna sinensis has been reported to inhibit VEGFA and suppress VEGFR1 and VEGFR2 and the phosphorylation of PI3K, FAK, Akt, and ERK1/2, which thereby inhibits angiogenesis and cancer growth [226]. Thymoquinone, a kind of monoterpene that controls metastasis and angiogenesis, is activated by suppressing the EGF, VEGF, and TGF-*β* pathways in the CRL-1739 cell line [227]. Emodin, an anthraquinone isolated from aloe vera, inhibits the proliferation of cancer cells by suppressing the expressions of MMP7, MMP9, VEGF, EMT, and the Wnt/*β*-catenin signaling pathway [228].

## 4. Future Direction

1. Many signaling pathways have been reported to be involved in the prevention and treatment of cancer, and the details of the associated mechanisms remain elusive
2. Future studies are required to elucidate the bioavailability and pharmacokinetics of these bioactive compounds
3. Although various studies have reported the positive effects of bioactive compounds in cancer treatments, the compounds from foods are not specific, and producing novel drugs based on the bioactive compound structures is important
4. Although the physiological effects of these bioactive compounds have been observed in animal models, there are currently few human clinical studies that evaluate these compounds. In the future, more clinical studies are essential to evaluate these compounds and to determine the optimal plasma levels
5. It is a great challenge to investigate individual compounds because the final effects are highly influenced by their interactions with each other
6. The anticancer effects of bioactive compounds vary in dose-dependent manners, and further research is needed to reveal the optimal doses or concentrations

## 5. Conclusion

Cancer is the leading cause of morbidity and mortality worldwide. It is urgent to develop more effective and specific treatment strategies to prevent cancer deterioration. Bioactive compounds have been identified as chemotherapeutic and chemopreventive agents in treating cancer. Furthermore, various pathways, such as ROS, ER stress, and epigenetic modifications, are reported to mediate the effect of bioactive compounds on cancer. Further experimental studies will be carried out to improve our understanding of the mechanisms related to the anticancer effects. This information may be helpful for the development of synergistic combinations to achieve better efficacy.

## Figures and Tables

**Table 1:** AICR's foods that fight cancer.

Natural products	Compounds	Strong evidence	Limited evidence
Apples	Dietary fiber, triterpenoid compounds, flavan-3-ols, flavonols	Colorectal cancer	Lung cancer

Asparagus	Flavonols, saponins, inulin, folate		Estrogen receptor-negative (ER-) breast cancer

Blueberries	Dietary fiber, flavones, vitamin C, flavan-3-ols, anthocyanins, tannins (proanthocyanidins and ellagitannins), flavonols, stilbenes, phenolic acids (mainly chlorogenic acids)	Colorectal cancer	Lung cancer

Broccoli and cruciferous vegetables	Glucosinolates, carotenoids (especially beta-carotene, lutein, and zeaxanthin)	Colorectal cancer	Lung cancer, ER- breast cancer, colon cancer

Brussels sprouts	Carotenoids, flavonols, dietary fiber, lignans, vitamin C, glucosinolates, folate	Colorectal cancer	Lung cancer, ER- breast cancer, colon cancer

Carrots	Carotenoids, phenolic acids (including chlorogenic acids), polyacetylenes (such as falcarinol)		Lung cancer, ER- breast cancer

Cauliflower	Vitamin C, folate, glucosinolates		Lung cancer, colon cancer

Cherries	Anthocyanins, beta-carotene, dietary fiber, melatonin, phenolic acids;flavan-3-ols, vitamin C, perillyl alcohol	Colorectal cancer	Lung cancer, colon cancer

Coffee	Melanoidins, lignans, phenolic acids (mainly chlorogenic acids), caffeine, diterpenes (cafestol and kahweol) in unfiltered coffee	Endometrial and liver cancers	Mouth, pharynx, larynx, and skin cancers

Cranberries	Anthocyanins, tannins, flavonols, terpenes, phenolic acids		Lung cancer

Flaxseed	Dietary fiber, gamma-tocopherol (a form of vitamin E), lignans, alpha-linolenic acid, phenolic acids	Colorectal cancer	

Garlic	Allium compounds		Colorectal cancer

Grapefruit	Vitamin C, flavanones, coumarins, carotenoids in pink and red grapefruit, terpenes in grapefruit peel		Lung cancer, esophageal cancer, stomach cancer, ER- breast cancer

Grapes	Flavonols, phenolic acids, resveratrol, flavan-3-ols, anthocyanins (in red and purple grapes)		Lung cancer

Kale	Dietary fiber, flavonols, folate, glucosinolates, carotenoids (especially beta-carotene, lutein, and zeaxanthin)	Colorectal cancer	ER- breast cancer, lung cancer, colon cancer

Oranges	Dietary fiber, flavanones, terpenes in orange peel, vitamin C	Colorectal cancer	Lung cancer, stomach cancer, colon cancer

Pulses: dry beans, peas, and lentils (legumes)	Dietary fiber, resistant starch, phenolic acids, flacan-3-ols, folate, anthocyanins (in red and black beans), tannins (especially proanthocyanidins), lignans, phytic acid saponins	Colorectal cancer	

Raspberries	Dietary fiber, ellagitannins, vitamin C, phenolic acids, anthocyanins	Colorectal cancer	Lung cancer

Soy	Isoflavones (genistein, daidzein, and glycitein), dietary fiber (whole soy foods), phytic acid, phenolic acids, protease inhibitors, folate, sphingolipids, lignans, saponins	Colorectal cancer	Lung cancer, breast cancer

Spinach	Carotenoids (especially beta-carotene, lutein, and zeaxanthin), flavonols, vitamin C, dietary fiber, folate, lignans	Colorectal cancer	ER- breast cancer, lung cancer, colon cancer

Squash (winter)	Vitamin C, carotenoids (beta-carotene, alpha-carotene, beta-cryptoxanthin, lutein, and zeaxanthin)		ER- breast cancer, lung cancer, colon cancer

Strawberries	Vitamin C, dietary fiber, anthocyanins, phenolic acids (including ellagic acid), stilbenes (mainly resveratrol), flavan-3-ols, tannins (proanthocyanidins and ellagitannins)	Colorectal cancer	Lung cancer, esophageal cancer, colon cancer

Tea	Flavan-3-ols, manganese, flavonols, caffeine, L-theanine		Bladder cancer

Tomatoes	Vitamin C, vitamin A, phytoene, phytofluene, lycopene, beta-carotene		ER- breast cancer, lung cancer, colon cancer, bladder cancer

Walnuts	Polyphenols, alpha-linolenic acid, phytosterols, melatonin, tannins (proanthocyanidins and ellagitannins)		

Whole grains	Dietary fiber, phytic acids, resistant starch, lignans, phenolic acids	Colorectal cancer	

**Table 2:** Several types of bioactive compounds.

Bioactive compounds	Examples	Sources	Supposed anticancer effects
Polyphenolic compounds	Quercetin, resveratrol, catechin	Red wine, chocolate, flaxseed oil	Carcinogen detoxification, inhibit tumor initiation/promotion, antimutagen

Carotenoids	Lycopene, lutein, cryptoxanthin	Tomatoes, carrots, leafy vegetables	Antimutagen

Sulfur-Containing compounds	Allicin, diallyl sulfide, allyl mercaptan Sulforaphane	Garlic, onion, leek	Carcinogen detoxification, inhibit tumor initiation/promotion

Terpenoid	Perillic acid, d-limonene	Cherries, mint, herbs	Carcinogen detoxification, inhibit tumor initiation/promotion

**Table 3:** Polyphenol classification.

Polyphenolic compounds	Sources	Examples	Chemical structure
Flavonoids	Chocolate, red wine, tea, apple, onions, broccoli, olives	Flavonols, flavones, flavanones, isoflavonoids, anthocyanidins, flavanols	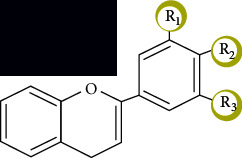
Phenolic acids	Coffee, black tea, cereals	Benzoic acid, cinnamic acid	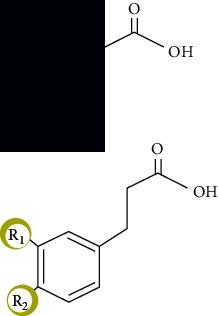
Stilbenes	Grapes, peanuts, red wine	Resveratrol	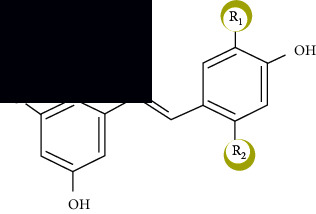
Lignans	Flaxseed oil, clover, lucerne	Secoisolariciresinol, matairesinol